# Molecular Characterization and Expression of *unc-13d* in the Sex Reversal of *Monopterus albus*

**DOI:** 10.3390/ani15020122

**Published:** 2025-01-07

**Authors:** Zitong Lian, Fang Meng, Xueping Xia, Junchao Fang, Haifeng Tian, Qiaomu Hu

**Affiliations:** 1 Yangtze River Fisheries Research Institute, Chinese Academy of Fishery Sciences, Wuhan 430223, China; 15621329336@163.com (Z.L.); 13659644863@163.com (F.M.); xiaxueping@biozeron.com (X.X.); 15119438380@163.com (J.F.); 2 State Key Laboratory of Mariculture Biobreeding and Sustainable Goods, Yellow Sea Fisheries Research Institute, Chinese Academy of Fishery Sciences, Qingdao 266071, China

**Keywords:** *Monopterus albus*, *unc-13d*, expression, in situ hybridization, DNA methylation

## Abstract

*Monopterus albus* is a protogynous hermaphroditic fish that changes from female to male, but the underlying sex change mechanism remains as-yet unknown. In this study, we firstly cloned and characterized the sequence and protein structure of *unc-13d* of *M. albus.* We found that the genomic structure of *unc-13d* was different from other species. Expression was detected in the developing gonad by applying qRT-PCR and in situ hybridization. We found that the expression of *unc-13d* in the ovotestis was higher than in the ovary and testes. A strong signal of *unc-13d* was detected in oocytes and granulosa cells in the ovary and spermatogonia and primary spermatocytes in the testes. We found that the promoter methylation of *unc-13d* was negatively correlated with gene expression in developing gonads, especially at site 114. A dual-luciferase assay was designed and revealed that *dmrt1* regulates promoter activity opposite to *foxl2*. In summary, during sex reversal, DNA methylation affects the binding of the transcription factor dmrt1 and *foxl2* in the promoter region through methylation and demethylation interactions to regulate the expression of *unc-13d* during gonadal development.

## Simple Summary

The aim of this research was to characterize the structure, expression, and function of *unc-13d* in the process of sex reversal in *Monopterus albus*. The expression profile of *unc-13d* suggested that *unc-13d* plays an important role in sex reversal. Additionally, the DNA methylation of the promoter of *unc-13d* exhibited negative correlation with gene expression, especially at site 114. Furthermore, we found that *dmrt1* antagonistically regulates *foxl2* expression through *unc-13d*.

## 1. Introduction

Sex determination is a complex process involving many different pathways and sex-related genes [1]. Sex determination mechanisms are generally categorized as genetic sex determination (GSD), which is common across the animal kingdom, and environmental sex determination (ESD), which has been found in diverse taxa, especially in teleost fish [2,3,4], and can be influenced by different environmental factors, such as temperature, hormones, pH, social conditions, and salinity [5]. Recent studies have confirmed that DNA methylation is an important epigenetic mechanism in sex transition [6,7,8]. For example, the epigenetic modification of ZW was found in *Cynoglossus semilaevis* when treated with high temperature [6], and increased methylation levels in the promoter of gonadal aromatase (*cyp19a*) were also observed in the ovaries of *Dicentrarchus labrax* at high temperatures [9].

*Monopterus albus* is widespread throughout East and Southeast Asia and is a highly favored food and commercially important fish in this region [10,11,12]. Interestingly, *M. albus* is a protogynous hermaphroditic fish that undergoes a sequential sex change from female to male after its first spawning [13,14,15,16,17]. This sex reversal process has attracted the attention of researchers around the world. Owing to its distinctive developmental sex traits and small genome, *M. albus* is expected to gradually become a new model species for research transformation and biological development [18,19,20,21]. Research on the regulation of sex in *M. albus* and its sex reversal mechanisms is increasing, and there have been a few studies analyzing the methylation-related factors that cause sex reversal in *M. albus* [22,23,24,25,26]. However, the precise role of DNA methylation in sex reversal in *M. albus* has not been fully clarified before now. Previously, we identified *unc-13d* as a candidate gene due to its low accessibility status, hypermethylation, and up-regulation of expression through DNA methylation upon the analysis of the gonads of female, male, and intersex specimens [26]. In this study, we cloned and characterized *unc-13d* of *M. albus*, analyzed its expression and localization in different gonadal stages, and further confirmed that its expression might be regulated by the binding of *dmrt1* in its promoter region, which might be regulated through methylation. The precise binding site of the transcription factor in the promoter region of *unc-13d* was also characterized using a dual-luciferase assay. This provides evidence supporting the role of *unc-13d* methylation in sex differentiation in *M. albus*.

## 2. Materials and Methods

### 2.1. Animal Specimen Collection and Sample Preparation

The *M. albus* specimens used in this study were acquired from the Breeding Center of Yangtze River Fisheries Research Institute, Chinese Academy of Fishery Sciences, China. Prior to sampling, twenty *M. albus* specimens were deprived of food for 24 h and anesthetized with MS-222 (500 mg/L, 5 min). Three sets of gonads were extracted from the *M. albus* groups, specifically female, intersex, and male, including at least three samples per group. The gonadal samples obtained from three *M. albus* specimens (aged one year) were separated into two parts: the first part was kept in 4% paraformaldehyde (pH = 7.5) for preparing tissue sections, and the second one was preserved in liquid nitrogen and then transferred to a −80 °C refrigerator for RNA and DNA extraction. Total DNA and RNA were extracted according to the manufacturer’s instructions, and the concentration was determined using an Agilent 2100 Bioanalyzer (Agilent Technologies, Santa Clara, CA, USA). Their integrity was confirmed through agarose gel electrophoresis.

### 2.2. Sequence Analysis and Construction of a Phylogenetic Tree

The open reading frames were predicted on the NCBI (http://www.ncbi.nlm.nih.gov/gorf/orfifig.cgi, accessed on 1 October 2024), and the domains were predicted using the InterPro database and SMART database [27] (http://www.ebi.ac.uk/interpro/; https://smart.embl-heidelberg.de/, accessed on 1 October 2024). The physicochemical properties of the *M. albus* UNC-13D protein were predicted using ProtParam [28] (https://www.expasy.org/resources/protparam, accessed on 1 October 2024). The secondary structure was analyzed using the online tool PSIPRED (http://bioinf.cs.ucl.ac.uk/psipred/, accessed on 1 October 2024), and the tertiary structure of *unc-13d* was predicted using the online tool SWISS-MODEL [29] (https://swissmodel.expasy.org/, accessed on 1 October 2024). The phylogenetic analysis was carried out using MEGA 7.0 software with the neighbor-joining (NJ) method [30]. Conserved amino acid sequences were predicted using the online tools WebLogo3 [31] and MEME [32] (http://weblogo.threeplusone.com/, accessed on 1 October 2024; https://meme-suite.org/meme/tools/meme, accessed on 1 October 2024). Genomicus [33] (https://www.genomicus.bio.ens.psl.eu/genomicus-108.01/cgi-bin/search.pl, accessed on 1 October 2024) databases were used to obtain the genomic arrangement and collinearity analysis of *unc-13d* in *Homo sapiens*, *Muroidea*, *Lepisosteus oculatus*, *Mastacembelus armatus*, and *M. albus*.

### 2.3. Real-Time Quantitative PCR

The expression of *unc-13d* at three gonad developmental stages was determined and analyzed by quantitative real-time fluorescence PCR (qRT-PCR). Total RNA was extracted from the gonadal samples using TRIzol (Invitrogen, Carlsbad, CA, USA), according to the manufacturer’s instructions. EF-1α was selected as the internal reference gene [34]. The primers used for the detection of *unc-13d* are given in Table 1. The qRT-PCR reactions were performed on a QuantStudio 5 real-time quantitative PCR system (Applied Biosystems, Carlsbad, CA, USA), as described previously [26]. The qPCR amplification was performed independently in three replicates of three samples per tissue. Relative expression was calculated by the selected 2^−ΔΔCT^ method [35]; data were analyzed by One-Way ANOVA and Duncan’s method of multiple comparisons using SPSS 22.0 (IBM, New York, NY, USA), and the significance was set at *p* < 0.05.

### 2.4. In Situ Hybridization

In order to assess the expression of *unc-13d* in gonadal cells, primers were designed using Primer Premier 5.0 design software [36]. The T7 promoter sequence was added to the 5′ end of the primers (Table 1). An 815 bp cDNA fragment of *unc-13d* was amplified, and the PCR products were purified and recovered using a Gel Recovery Kit (TIANamp) according to the manufacturer’s instructions. RNA integrity and concentration were detected using 1% agarose gel and a Nano Photometer NP80 (Implen, München, Germany), and the probes were prepared with a MEGAshortscript T7 High Yield Transcription Kit (Invitrogen, AM1354). In situ hybridization was performed as follows: Sections were deparaffinized, hydrated, and treated with proteinase K, and then hybridized using a sense or antisense DIG-labeled RNA probe at 70 °C for 12 h. Hybridization signals were then detected with anti-DIG (Roche, 11093274910) conjugated with POD. DAB (Beyotime, Nantong, China, P0202) was used as the substrate for POD.

### 2.5. Methylation State Change of unc-13d Promoter During Sex Reversal

To determine the methylation status of the promoter of *unc-13d* in different gonadal stages, a 1500 bp promoter was obtained from the genomic database and a 602 bp methylation island was predicted. The genomic DNA of at least 15 samples in one group was extracted. At least 2 μg of mixed DNA was treated using the DNA methylation kit (Zymo, CA, USA) according to the manufacturer’s instructions. Primers were designed using MethPrimer [37] (http://www.urogene.org/methprimer/, accessed on 1 October 2024). The treated DNA was used as a template for PCR amplification. The PCR system consisted of 0.125 µL TaKaRa Ex Taq HS (Takara, Dalian, China), 2.5 µL 10 × Ex Taq Buffer, 2 µL dNTP Mixture, 100 ng treated genomic DNA, 0.5 µL of each primer (10 μmol/L), and double-distilled water to reach a final volume of 25 μL. The reaction conditions were as follows: 98 °C for 10 min; 33 cycles of 98 °C for 10 s; 55 °C for 30 s; 72 °C for 1 min; and 4 °C for storage. PCR products were extracted, purified, and then cloned into the PMD-18T vector (Takara, Dalian, China). Each set of methylated sequences was sequenced for 15–20 positive clones, and methylation analysis was performed using the DNA analysis platform [38] (http://services.ibc.uni-stuttgart.de/BDPC/BISMA/, accessed on 1 October 2024) to compare the ratio of methylated to unmethylated CpG at each site in each tissue. The differences in mean methylation levels between groups were determined using independent samples t-tests. The differences in the proportion of methylated and unmethylated CpG at each locus were tested by the Chi-square test followed by Fisher’s exact test. A *p*-value of less than 0.05 was considered significant.

### 2.6. Plasmid Construction

The putative promoter region of *unc-13d* was obtained from the genomic database and the sex-related transcription factor binding sites were detected using JASPAR online software [39]. Three deletion fragments (2057, 1596, and 1008 bp) of the promoter were amplified from the genomic DNA, and the PCR products were purified and cloned in the PGL3-basic vector using KpnI and XhoI restriction endonucleases [40,41,42]. The fixed-point mutagenesis of the *dmrt1* and *foxl2* binding sites was performed using a Fast Site-Directed Mutagenesis Kit (TIANGEN, Beijing, China), according to the manufacturer’s instruction.

### 2.7. Cell Culture and Dual-Luciferase Assay

HEK293T cells were obtained from the Center of Animal Science and Animal Medicine, Shandong Agricultural University, cultured in 24-well plates, and incubated with DMEM complete medium (10% double antibodies, 10% fetal bovine serum, and DMEM high sugar medium) at 37 °C in a 5% CO_2_ incubator to reach a cell density of 50%. The DMEM (Termo Fisher Scientifc, Waltham, MA, USA) was replaced with opti-MEM medium (Termo Fisher Scientifc, Waltham, MA, USA) and transfected with a 50:50:1 ratio of recombinant constructs to PGL3-promoter vector to sea kidney luciferase vector using Lipofectamine^TM^ 3000 (Invitrogen, Carlsbad, CA, USA), according to the manufacturer’s instructions, and then incubated at 37 °C for 6 h. Subsequently, the opti-MEM medium was removed and the cells were incubated for 48 h in DMEM. The cells were collected, the luciferase activity was measured using a dual-luciferase kit (Promega, Madison, WI, USA), and the fluorescence intensity was measured using a BHP9504 microplate chemiluminescence immunoassay reader (Hamamatsu, Shizuoka City, Japan) with a determination interval of 2 s and a determination time of 2 s.

## 3. Results

### 3.1. Sequence Analysis of unc-13d

**Figure 1:**
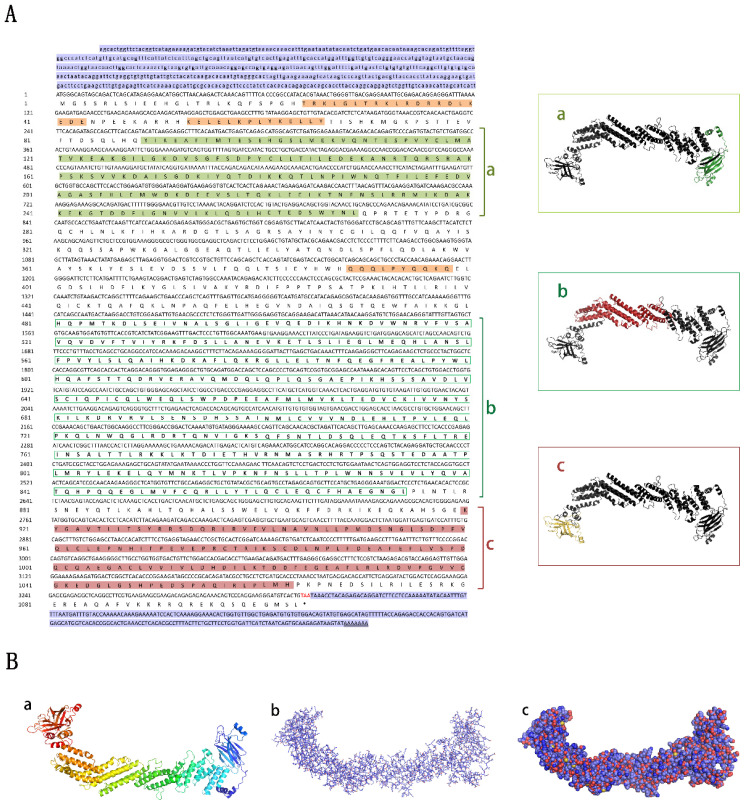
Nucleotide sequence of *Monopterus albus unc-13d* cDNA and its protein 3D structure. (**A**) Nucleotide sequence and deduced amino acid sequence of UNC13D. a: The green region is the C2A-Munc13-like conserved domain. b: Green squares are C2B-Munc13-like conserved domains. c: Red region is the DUF 1041 super family conserved domain. The orange regions are the three low-complexity domains. (**B**) UNC13D protein model. (**a**) Model of UNC13D protein cartoon ribbons; (**b**) UNC13D protein stick model; (**c**) UNC13D protein sphere model.

With the help of RACE techniques, the full-length cDNA (4455 bp) of the *unc-13d* of *M. albus* (Genbank No: XP020481010), including 5′UTR (626 bp) and 3′UTR (257 bp), was obtained for the first time. The predicted open reading frame (ORF) was 3315 bp, encoding 1105 aa (Figure 1A). The molecular formula of UNC-13D was C_5620_H_8904_N_1550_O_1693_S_38_, and the relative molecular mass was 126.348 kDa. The instability coefficient was 45.29—greater than 40—the lipid coefficient was 87.24, and the total average hydrophilicity was −0.501, suggesting that it is an unstable hydrophilic protein. The secondary structure of the protein contains 52.08% α-helix, 9.96% β-turn, and 37.95% irregular curl. The predicted tertiary structure of the UNC-13D protein is presented in Figure 1B. Its Global Model Quality Estimate (GMQE) score is 0.54 (between 0 and 1), indicating that the predicted tertiary structure model is highly accurate, and the estimated QMEAN score is −3.56 (between 0 and −4), suggesting that the predicted model structure has good consistency. A combination of Smart database analysis and the online tool CD-Search showed that the tertiary structure of the protein contains three low-complexity regions (LCRs) (Figure 1A): the C2A-Munc13-like conserved domain (C2 domain first repeat in Munc13 in the amino acid region from position 89 to 269 (Figure 1A); the C2B-Munc13-like conserved domain (C2 domain second repeat in Munc13 in the amino acid region from position 920 to 1063 (Figure 1A); and the DUF 1041 superfamily conserved domain in the amino acid region from position 481 to 874 (Figure 1A).

**Figure 2:**
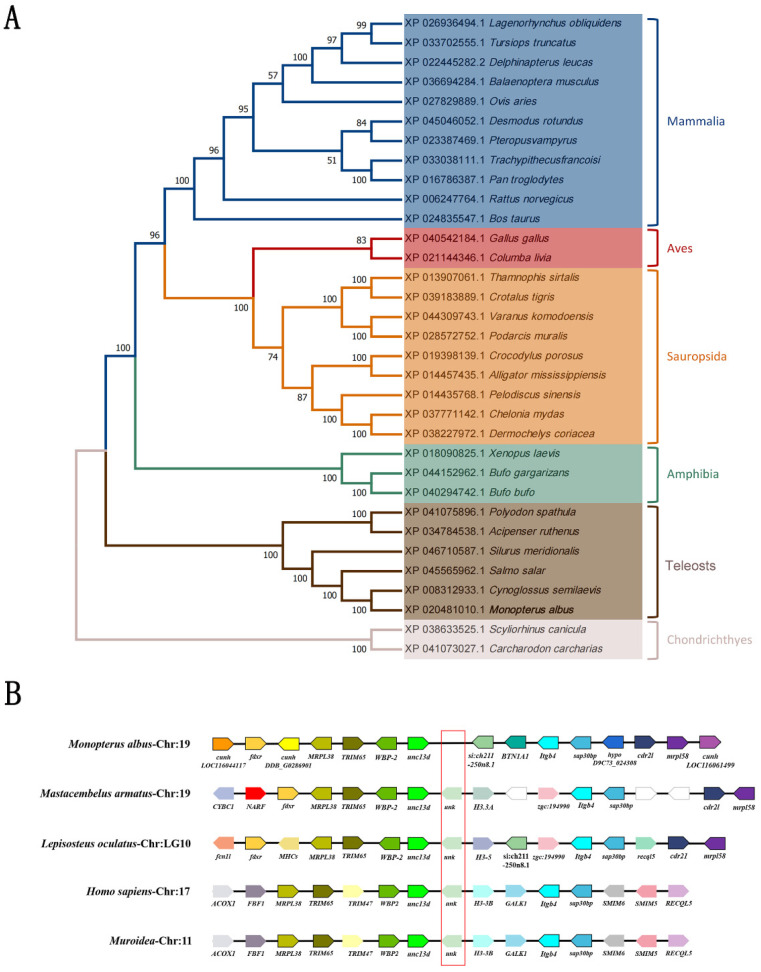
Phylogenetic tree of the amino acid sequence and gene structure of UNC13D. (**A**) Phylogenetic tree of the amino acid sequence of UNC13D; (**B**) gene structure of *unc-13d* among species.

On the obtained NJ tree, homologs of mammals, birds, and reptiles were grouped separately, homologs of cartilaginous fishes were clustered on a separate branch, and the *unc-13d* of *M. albus* was grouped with the homolog of the half-smooth tongue sole (*Cynoglossus semilaevis*). Such phylogenetic topology is generally consistent with the known evolutionary pattern of the species (Figure 2A). Using Genomicus, a conserved co-linear gene block of *unc13-d*, including *WBP-2*, *TRLM65*, *MRPL38*, and *fdxr* in the upstream region and *Itgb4*, *sap30bp*, *cdr21*, and *mrpl58* in the downstream region, was found in different vertebrate species and even in mammals (Figure 2B). This conserved co-linearity of *unc-13d* and its neighboring gene blocks suggests the conservation of the function of *unc-13d* among vertebrates.

### 3.2. unc-13d Expression Associated with DNA Methylation During Gonadal Development

**Figure 3:**
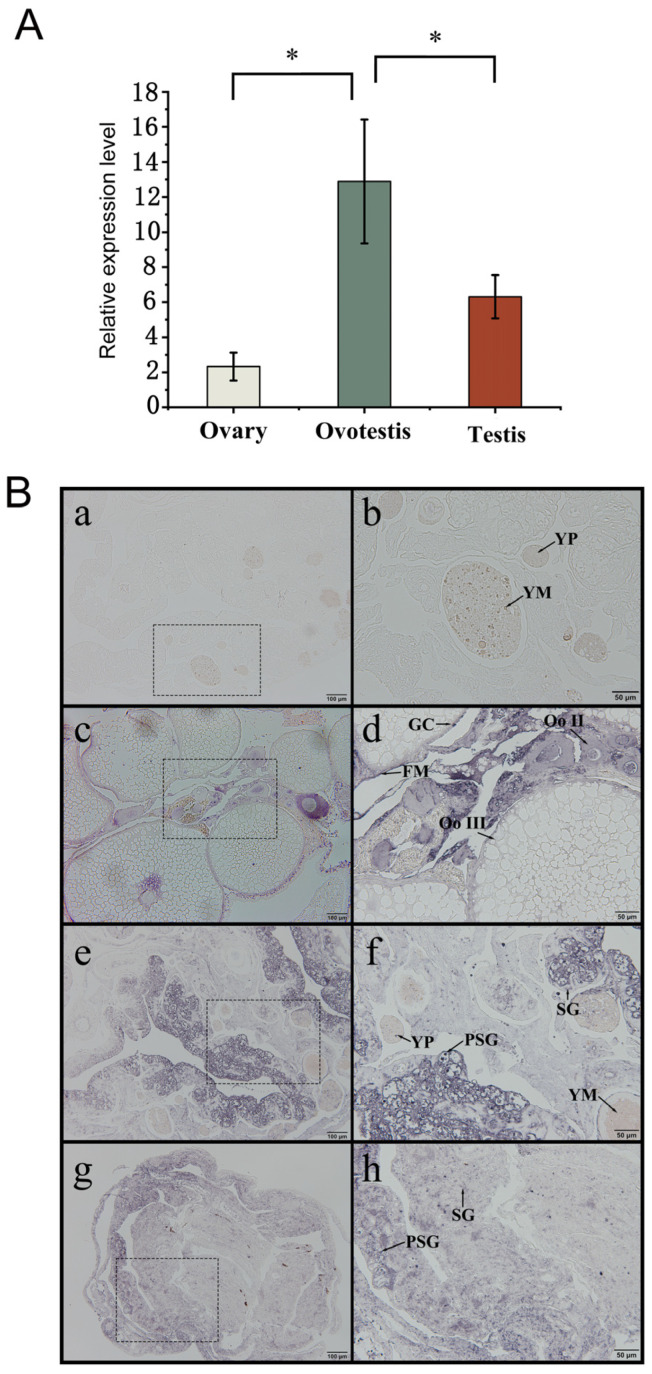
Expression and localization of *unc-3d* during gonad development in the ovaries, ovotestes, and testes of the *Monopterus albus*. (**A**) Detection of *unc-3d* expression in ovaries, ovotestes, and testes by qRT-PCR. (**B**) Detection of *unc-3d* RNA expression in ovaries, ovotestes, and testes by in situ hybridization. FM: Follicular membrane; Nu: Nucleolus; Oo II: phase II oocytes; Oo III: phase III oocytes; PSG: primary spermatogonia; SG: spermatogonia; YM: yolk mass; Yp: yolk platelet; GC: granulosa cells; (**a**,**b**): control without probe; (**c**,**d**): ovary; (**e**,**f**): ovotestis; (**g**,**h**): male; (**b**,**d**,**f**,**h**) show the large magnification of frame areas in (**a**,**c**,**e**,**g**), respectively. The asterisk (*) indicates a significant difference between the two groups.

**Figure 4:**
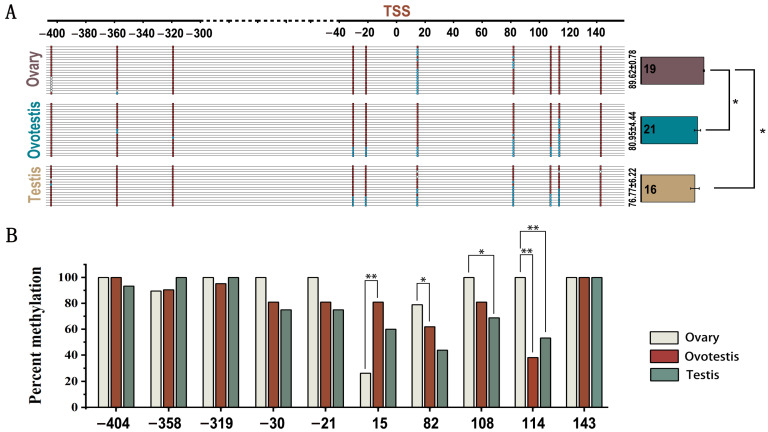
Changes of *unc-13d* promoter methylation levels during sex reversal. (**A**) Methylation level changes during sex reversal process. The red, blue, and white boxes indicate methylated, unmethylated, and unknown positions, respectively. Results are presented as mean ± SE. (**B**) The percentage of DNA methylated at the CpG site. An asterisk (*) indicates a significant difference between the two groups, while two asterisks (**) indicate a highly significant difference.

The expression of *unc-13d* was significantly higher in the ovotestes than in the ovaries and testes (Figure 3A, *p* < 0.05). No signal was detected in the control group (Figure 3(Ba,b)). A strong signal of *unc-13d* was detected in the cytoplasm and granulosa cells of oocytes (Figure 3(Bc,d)). In the ovotestes and testes, *unc-13d* expression was mainly detected in the spermatogonia and primary spermatocytes (Figure 3(Be–h)). To test the mechanism of the different expression of *unc-13d* during sex reversal, the methylation island of the *unc-13d* promoter was analyzed through MethPrimer software and a primer was designed. The methylation status of each locus at different gonadal developmental stages was examined. The results showed that the level of methylation of gonadal development decreased from 89.62 in the ovaries to 76.77 in the testes (Figure 4A). The results of each locus exhibited that sites 15, 82, 108, and 114 had significant differences in methylation levels during sex reversal. However, only at site 114 was methylation level was negatively associated with gene expression (Figure 4B).

### 3.3. Dmrt1 Regulates Promoter Activity Opposite to FoxL2

**Figure 5:**
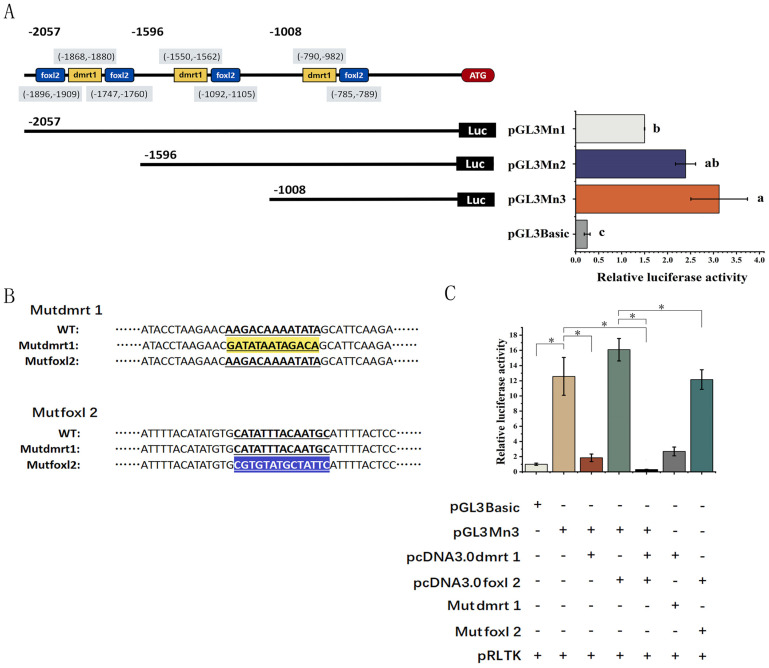
Luciferase detection of transcription factor binding sites and activation. (**A**) Schematic diagram of *dmrt1* and *foxl2* binding sites. (**A**) Schematic diagram of deletion fragments in the promoter region of *unc-13d* in *Monopterus albus.* (**B**) Schematic diagram of the DNA sequences of the mutant and wild type. The dmrt1 and *foxl2* mutation sites are shown in yellow and blue, respectively. (**C**) Luciferase assay reveals *unc-13d* promoter activity in 293T cell. The mean ± SEM was from three independent experiments; * *p* < 0.05 shows significant difference. Groups with different letters are significantly different (*p* < 0.05).

By analyzing potential binding sites for transcription factors in the promoter region, several sex-related transcription factor binding sites were found using the JASPAR online software (Figure 5A). The pre-2057 bp of the transcription start site (TSS) was selected as a candidate promoter region. For the accurate localization of the binding sites, a PGL3 dual-luciferase reporter assay with a series of deletions of the *unc-13d* promoter region was constructed. The results showed that luciferase activity was significantly higher (*p* < 0.05) in the three deletion constructs than in the basic group (Figure 5A). The luciferase activities indicated that key regulatory elements are distributed within the first 1008 bp of the transcription start site, including *dmrt1* and the *foxl2* binding site (Figure 5A).

To further explore the specific role of the sex-linked transcription factors *dmrt1* and *foxl2* in the *unc-13d* promoter, we constructed site-specific mutants using the PGL3-Mn3 plasmid as a template (Figure 5B). Two mutants, Mut-dmrt1 and Mut-foxl2, were obtained (Figure 5B). Luciferase activities were significantly reduced after pcDNA3.0-dmrt1 was used together with pGL3-Mn3 (*p* < 0.05, Figure 5C), indicating that *dmrt1* inhibited the activation of the *unc-13d* promoter. Additionally, we found that the *foxl2* binding site mutation caused a significant down-regulation of the luciferase activity in the Mut-foxl2  +  pcDNA3.0-foxl2 group (*p*  <  0.05) (*p*  >  0.05, Figure 5C). These findings suggested that dmrt1 inhibited the activation of the *unc-13d* promoter, while *foxl2* increased its activation.

## 4. Discussion

DNA methylation play crucial roles in sex determination in animals [6,7,8,9]. As a protogynous hermaphroditic fish, *M. albus* can transform its gender after its first spawning [13,14], and epigenetic modifications are considered to be among the potential mechanisms for this sex transformation process [25,26].

*Unc-13d* has been found to be a candidate gene for the sexual transformation of *M. albus* due to its hypermethylated promoter and low accessibility status, as well as its up-regulation in our previous methylome study [26]. *unc-13* has been revealed to be involved in vesicle transport, vesicle guidance, anchoring, and release processes [43,44,45]. *unc-13d* was first identified in hematopoietic cells [43], and was believed to function as a regulator of the secretory organelle cytosolic emesis initially [46,47]. *unc-13b*, one homolog of *unc13*, has been characterized as a potential marker for male fertility in pig and boars [48,49]. However, the physiological roles of *unc-13d* in the sex differentiation process remain unclear. In this study, the full-length cDNA sequence of *unc-13d* of *M. albus* with 4455 bp was obtained. The structural domain analysis indicated that the *unc-13d* of *M. albus* contains three conserved regions: C2A, C2B, and DUF1041. On the obtained phylogenetic evolutionary tree, the characterized *unc-13d* was recovered to a group with homologs from half-smooth tongue sole; homologs from Cartilaginous fish and those of mammals, reptiles, and birds formed separate clades, which is generally consistent with the general pattern of animal evolution. These findings suggest that the evolution of *unc-13d* is conservative across different groups. The collinearity analysis of *unc-13d* and its neighboring genes (Figure 2B) revealed that the co-linear block of *unc-13d* is highly conserved between *Lepisosteus oculatus* and *Mastacembelus armatus*, and even among mammals, with some exceptions in the gene orientation of some neighboring genes. All these findings suggest that the characterized *unc-13d* is conserved among vertebrates.

Using qRT-PCR, *unc-13d* expression was found to be significantly higher in the ovotestes than in the ovary and testes. The expression localization analysis highlighted that *unc-13d* was predominantly expressed in oocyte cytoplasm and granulosa cells in the ovary, as well as the spermatogonia and primary spermatocytes in the ovotestes and testes.

Methylation levels were considerably lower at site 114 in the ovotestes compared to the ovaries and testes, and a negative correlation between *unc-13d* expression and its promoter methylation was found. In the promoter region, pre-2057 bp was identified as a candidate region for transcription factor binding. To accurately locate the promoter binding site, activation of the promoter was detected in different regions of the promoter. *Dmrt1* is a crucial player in male sexual development across mammals, birds, amphibians, and scleractinians [50], and *foxl2* is recognized as a highly conserved gene across vertebrates, with involvement in almost all stages of ovarian development. The function of *foxl2* in ovarian development has been determined in several species [42,51,52,53,54,55,56]. Site-specific mutants were constructed to pinpoint the exact binding site for the expression of *unc-13d* during transcription-factor-mediated sex reversal, utilizing the PGL3 plasmid as a template. The findings demonstrate a marked reduction in the relative fluorescence intensity of dmrt1 subsequent to the mutation of the dmrt1 binding site, thereby providing evidence of dmrt1’s ability to engage with the UNC-13D promoter and catalyze the expression of *unc-13d*. To summarize, DNA methylesterases influence the *dmrt1* transcription factor’s binding to the promoter region via methylation interactions, leading to alterations in *unc-13d* function during sexual transformation.

## 5. Conclusions

*M*. *albus* is a hermaphroditic fish that changes from female to male, but its underlying sex reversal mechanism remains unknown. The present study characterized the structure, expression, and function of *unc-13d* in the process of sex reversal in *M. albus*. We found that the genomic structure of *unc-13d* was different from other species. The highest expression was detected in the ovotestes and a strong signal was detected in oocytes and granulosa cells in the ovaries and spermatogonia and primary spermatocytes in the testes, suggesting that *unc-13d* plays an important role in sex reversal. Additionally, the DNA methylation of the promoter of *unc-13d* exhibited a negative correlation with gene expression, especially at site 114. Finally, we found that *dmrt1* and *foxl2* antagonistically regulate *unc-13d* expression. Taken together, we infer that during sex reversal, DNA methylation affects the binding of the transcription factors dmrt1 and *foxl2* in the promoter region through methylation and demethylation interactions to regulate the expression of *unc-13d* during gonadal development.

## Figures and Tables

**Table 1:** Primers used in this study.

Primer	Primer Sequences (5′-3′)	Utilization	Products Size
Unc13d-5′	TATTGTTGTTTAATGGCGATG	5′RACE amplification	/
Unc13d-3′	ACCGCAAATACCACCACA	3′RACE amplification	/
Unc13d-S1	TTGACGAGGAAATTGCGAGAC	In situ hybridization	815 bp
Unc13d-S-T7	GATCACTAATACGACTCACTATA GTTGACGAGGAAATTGCGAGAC	In situ hybridization	815 bp
Unc13d-A-T7	GATCACTAATACGACTCACTATA GCCAGCACTCAGCGTCCCAT	In situ hybridization	815 bp
Unc13d-A1	CCAGCACTCAGCGTCCCAT	In situ hybridization	815 bp
Unc13d-S	TTGACGAGGAAATTGCGAGAC	qRT-PCR	148 bp
Unc13d-A	TTGTTGACGGTTTACCCATCTTAT	qRT-PCR	148 bp
EF-1α-F	CGCTGCTGTTTCCTTCGTCC	Internal reference	102 bp
EF-1α-R	TTGCGTTCAATCTTCCATCC	Internal reference	102 bp

## Data Availability

The data presented in this study are available in the article.
